# Intravenous Ketamine to Facilitate Transport of Agitated Patients to the ECT Clinic

**DOI:** 10.1097/YCT.0000000000001090

**Published:** 2024-11-26

**Authors:** Olivia Dean, Anthony Byford-Brooks, Kara Hannigan, Danielle Saunders, William Gamble, George Kirov

**Affiliations:** From the ∗ ECT Clinic, Hafan y Coed, University Hospital Llandough, Llandough, United Kingdom; † Department of Anaesthetics, University Hospital of Wales, Cardiff, United Kingdom; ‡ Centre for Neuropsychiatric Genetics & Genomics, Division of Psychological Medicine and Clinical Neuroscience, Cardiff University School of Medicine, Cardiff, United Kingdom.

**Keywords:** ECT, ketamine, transfer, sedation, psychosis

## Abstract

**Objectives:**

Electroconvulsive therapy (ECT) can be effective for a variety of psychiatric conditions, including for some patients who are very psychotic or agitated. Transferring such patients from the psychiatric ward to the ECT clinic can pose significant challenges for treating teams, as they try to minimize the use of restraint.

**Methods:**

We developed a protocol for safe transfer of such patients using sedation with ketamine. An intravenous cannula is inserted on the ward in a low stimulus environment with gentle supportive holds. Intravenous ketamine is given in a bolus at 0.5–2.0 mg/kg. The patient is transported on a transfer bed to the ECT clinic within a few minutes, and usual ECT process is immediately followed.

**Results:**

We describe 6 patients who were given between 1 and 11 ECT treatments using this method. All of them finished ECT courses without the need for ketamine sedation. Five of them regained capacity, provided informed consent for further ECTs, and eventually reached remission.

**Conclusions:**

Ketamine can be used to manage risk and transfer agitated patients to an ECT clinic for treatment.

Since its development over 80 years ago, electroconvulsive therapy (ECT) remains a well-established and effective treatment for moderate-to-severe depression.^1^ Though primarily prescribed for use in depressed patients in Western countries, ECT can also be administered to patients who have a diagnosis of schizophrenia or mania.^2^ Significant challenges can sometimes be posed by severely unwell patients who are agitated or disturbed as a result of their illness and lack capacity to understand the necessity for treatment. Restraint can be used to facilitate treatment, but this can risk injury to both staff and patients, as well as cause a traumatic experience to unwell patients.

We received several referrals for extremely unwell patients for whom transfer to the ECT clinic posed a challenge and could result in prolonged restraint because they lacked capacity and were uncooperative or severely agitated. We considered various options of how this could be facilitated safely, minimizing the use of restraint and distress to the patient.

Within psychiatry, the aim of rapid tranquillization (RT) should not be to completely sedate initially. Instead, the aim should be to help the patient to calm down.^3^ The local policy for RT of an agitated patient within Cardiff and Vale University Health Board suggests the use of lorazepam in the first instance. Promethazine is then suggested as second-line RT and finally the use of antipsychotic medication (haloperidol or olanzapine) as third-line RT. Five of our 6 patients were already nursed in a psychiatric intensive care unit (PICU) and were prescribed high doses of antipsychotic and benzodiazepine medications. Therefore, additional doses would not have provided a sufficient effect. They would have also required restraint for the medication to be administered and may have had to remain in restraint due to potential risks to themselves and others. This is likely to increase risk of not only physical injury to the patient and staff but also of psychological injury.^4^ Additionally, benzodiazepines can reduce the quality of the seizure induced during ECT.^5^ Consideration was also given to administering general anesthetic on the PICU using propofol and then transferring the patient to the clinic. However, our anesthetic colleagues deemed this to be unsafe due to the potential of suppression of breathing during the transfer between the PICU and the clinic, which required the team to go in an elevator.

We discussed the problem with colleagues in other ECT clinics. One option suggested was a protocol for the administration of intramuscular ketamine to transfer patients (Oliver Cramer, personal communication, more details below).

Ketamine is a widely available anesthetic agent. It gained Food and Drug Administration approval in 1970 and was first given to American soldiers during the Vietnam War. It induces a state of “dissociative anesthesia”—providing pain relief, sedation, and amnesia to the patient.^6^ Ketamine suppresses breathing much less than other anesthetics—such as the ones usually administered during ECT—and allows patients to maintain their own airway.^7^ Ketamine is also superior to benzodiazepines like midazolam for sedation because it has much less of a negative impact on seizure quality.^8^ Because of these characteristics, ketamine could be a useful medication for transferring complex patients to an ECT clinic for treatment.

Ketamine has been used to facilitate the transfer of acutely agitated patients in various circumstances, not related to ECT. Le Cong et al^9^ describe a case study of an acutely agitated pregnant lady who needed to be transferred to hospital via air ambulance in remote Western Australia. Ketamine was administered in this instance to safely sedate and transfer the patient, negating the need for general anesthesia and tracheal intubation, which would have had increased risks due to the pregnancy. Brendt et al^10^ published a case study of a 19-year-old patient with a diagnosis of autism, social anxiety, agoraphobia, and morbid obesity who required transport from one residential unit to another. They administered intramuscular (IM) ketamine to sedate the patient before giving additional propofol to facilitate the transfer safely. Le Cong and Humble^11^ published a systematic review looking at transferring acutely agitated patients via air ambulance in Australia. Their findings indicate that using ketamine as sedation reduced the number of intubations in this patient group.

There is a scarcity of information on how to sedate aggressive or agitated patients prior to ECT. Smith-Martinez et al^12^ discuss the case of a 70-year-old lady with a diagnosis of schizoaffective disorder. They administered a subanesthetic dose of IM ketamine to the patient to safely sedate her in order for ECT to be administered. Oliver Cramer's protocol from the Isle of Wight NHS Trust, United Kingdom, describes a process whereby IM ketamine is administered to the thigh of a patient to quickly sedate them for transfer for ECT. Once the patient is sedated, the team will then gain intravenous (IV) access, secure the airway and apply bag valve mask ventilation (if required), attach monitoring, and then transfer the patient to the ECT clinic on a trolley for treatment. The protocol outlines that the full effect of the IM ketamine should be observed within 3–5 minutes. We reasoned that the IM route will leave the patient under the influence of ketamine for a prolonged time, beyond the duration of the ECT, and will allow less flexibility with the dose. Our anesthetic colleagues suggested to use an IV route instead. The use of IM ketamine remained as a “plan B” in situations where the team cannot establish an IV access.

## METHODS

We considered a few practical issues when using this method to transfer patients. The priority is to limit distress to the patients when transferring them to the clinic. The patient should ideally be the first on the list to be treated that day, and all other patients are scheduled to arrive later to maximize staff availability during the transfer. A robust handover should be held prior to each treatment session with all members of the participating team, with clear roles allocated. The route from the ward to the clinic should be planned in advance. If an elevator is to be used, that should be checked prior to transfer to ensure it is in full working order.

A patient transfer bed with wheels, side rails, and underbed storage was used to transfer the equipment and then the patient. A comprehensive list of equipment was composed to minimize the risk of errors or omissions (Table 1). An emergency grab bag of essential equipment (such as airway adjuncts, bag valve mask) was also available in the event of emergency. A portable suction machine and defibrillator were stored under the bed. Oxygen via a portable cylinder and face mask was also available. Portable monitoring, including 3-lead electrocardiogram, pulse oximetry, and blood pressure monitoring, was also used during transfers. CO_2_ monitoring should be available, as per the Association of Anesthetists of Great Britain and Ireland guidelines.^13^

**TABLE 1:** Equipment List Required for Ketamine Transfer

Under the trolley	Portable suction Oxygen cylinder Face mask with green tubing, attached to cylinder Water circuit with anesthetic mask
On the trolley	Scoop stretcher Portable patient monitor with capnography attached Extra oxygen cylinder Emergency bag—set with patient specific equipment (ie, size 6 tubes, size 3 supraglottic airway)
Emergency medication bag	• Glycopyrronium bromide • Atropine sulfate • Metaraminol • Ephedrine hydrochloride
Ketamine bag	• 5 mL Ketamine (100 mg) with green needle for IM • 10 mL Ketamine (10 mg/mL) IV • 20 mL Saline flush
Muscle relaxant + propofol bag	• Suxamethonium chloride • 20 mL propofol • 50 mg rocuronium bromide • Additional 20 mL ketamine (10 mg/mL)
Cannulation tray	• 10 mL saline flush • 4× 22G cannulas • 2× IV dressings • 2× Alcohol wipes • Tourniquet

The wards were contacted in advance of the team's arrival, and the patient was encouraged to sit in a low-stimulus room with nursing staff who have a good rapport with them. Often music was played to help calm the patient, and we found that this had a very positive effect. Nursing staff would sit either side of the patient on a sofa, using gentle supportive holds while the anesthetist and the operating department practitioner attempt to insert a cannula for the ketamine to be administered.

This process can take some time, and multiple attempts to approach the patient may be required. Distraction and encouragement techniques are key to facilitate this essential process. Once the cannula is in situ and flushed with saline, IV ketamine can be administered in doses of 0.5–2 mg/kg depending on patient's response. The patient can then be supported from a seated position on the sofa to the transfer trolley and encouraged to lie down. Monitoring is then applied, and oxygen was administered through face mask. Once the patient is settled on the transfer trolley, they can then be transferred to the ECT clinic for treatment. Additional bolus doses of ketamine may be administered on the way to the clinic to ensure the patient remains sedated throughout the transfer.

Staff who are trained in restraint techniques must be with the patient at all times, and the medication tray and the emergency equipment bag must go in the lift with the patient. Once in the treatment room, ECT can proceed as usual, starting with the World Health Organization Surgical Safety Checklist, adapted for ECT. After that, the short-acting anesthetic for ECT anesthesia (eg, propofol or methohexital) and muscle relaxant can be administered. After treatment, recovery can proceed as normal; however, this can be more prolonged when ketamine has been used.

## RESULTS AND DISCUSSION

The goal of this method is to safely transfer the patient for treatment while limiting the use of restraint and distress caused to the patient. The ultimate goal is for the patient to recover from their illness sufficiently and to be able to attend the clinic for ECT without the use of sedation. This method was used with a total of 6 patients aged between early 20s to late 70s (Table 2).

**TABLE 2:** Details on the Patients Treated With This Method

Patient ID	Diagnosis	No. ECTs With Ketamine	Dose of Ketamine	2**^nd^** Anesthetic	Total No. ECTs	BPRS Start	BPRS End	Outcome
A	Acute mixed affective/psychotic episode	7	0.5–1.3 mg/kg (30–80 mg)	Methohexital	14	89	27	Remission
B	Acute psychotic episode	1	1 mg/kg (75 mg)	Methohexital	7	65	24	Remission
C	Acute psychotic episode	1	1 mg/kg (70 mg)	Methohexital	9	65	24	Remission
D	Acute psychotic episode	11	1–2 mg/kg (65–140 mg)	Methohexital/etomidate	16	103	24	Remission
E	Schizophrenia	6	0.5–1 mg/kg (40–80 mg)	Propofol	9	92	76	Symptoms improved
F	Psychotic depression	4	0.6–1.2 mg/kg (40–80 mg)	Etomidate	12	HAM 41	HAM 3	Remission

BPRS, Brief Psychiatric Rating Scale; HAM, Hamilton Depression Rating Scale, 24-Item Version.

There were no complications noted during any of the transfers. Five out of the 6 patients achieved remission with ECT. Patient E was experiencing distressing psychotic symptoms, which improved with ECT, and although no remission was achieved, the patient was able to be discharged from PICU to a mental health rehabilitation ward.

Patients B and C only required 1 ECT session each using sedation for transfer before they improved enough to attend for further sessions without the use of sedation. In the cases of patients A and D, who required repeated transfers under sedation, progressively higher doses of ketamine were needed to ensure the patient remained sufficiently sedated to transfer.

Although no complications were noted in this study, treating teams should be mindful of the risks associated with this method. There is a risk of developing distressing psychedelic/dissociative symptoms in already psychotic patients. Care should be taken by the treating team that a sufficient dose of ketamine is administered so the patient remains sedated and does not experience distressing symptoms. Interviews with patients in this study after they had reached remission did not indicate any distressing experiences during the transfer. If the treating team feels that patients are experiencing distressing psychedelic symptoms, then they should pause to review whether this is the most appropriate method for them.

There are common side effects of ketamine that require careful monitoring. These include diplopia, confusion, tachycardia, agitation, excess secretions, and oversedation. Nystagmus was also observed in the case of 1 patient during 1 episode that required higher doses of ketamine; however, this resolved when the effects of the ketamine wore off.

Another advantage with using ketamine is the reduced impact on seizure threshold compared with other sedatives such as midazolam.^8^ However, it is important to not become overreliant on using sedation to transfer patients and only resort to it when it is assessed as safe and appropriate. High-quality risk assessments are key here. As patients' symptoms improve with ECT, the use of ketamine to transfer them should stop.

## CONCLUSIONS

Our initial experience showed that IV ketamine can be used in certain situations to manage risk and transfer agitated patients to an ECT clinic for treatment. The team should take careful consideration of the risks and benefits of this method. Good quality risk assessments, communication, and planning are key for delivering patient-centered care. As patients' symptoms improve, there should be no further need for sedation for transfer.
